# Nanoindentation Reveals Crosslinking Behavior of Solar Encapsulants—The Methodological Advantages over Bulk Methods

**DOI:** 10.3390/polym13193328

**Published:** 2021-09-29

**Authors:** Djamel Eddine Mansour, Christoph Herzog, Petra Christöfl, Luciana Pitta Bauermann, Gernot Oreski, Andreas Schuler, Daniel Philipp, Paul Gebhardt

**Affiliations:** 1 Fraunhofer Institute for Solar Energy Systems ISE, 79110 Freiburg im Breisgau, Germany; djamel.eddine.mansour@ise.fraunhofer.de (D.E.M.); christoph.herzog@ise.fraunhofer.de (C.H.); luciana.pitta.bauermann@ise.fraunhofer.de (L.P.B.); daniel.philipp@ise.fraunhofer.de (D.P.); 2 Laboratoire d’Energie Solaire et Physique du Bâtiment (LESO PB), Ecole Polytechnique Fédérale de Lausanne (EPFL), Station 18, 1015 Lausanne, Switzerland; andreas.schueler@epfl.ch; 3 Polymer Competence Center Leoben GmbH, AT-8700 Leoben, Austria; petra.christoefl@pccl.at (P.C.); gernot.oreski@pccl.at (G.O.)

**Keywords:** nanoindentation, encapsulant, degree of curing, creep measurement, frequency sweep measurement, dynamic mechanical analysis

## Abstract

The power degradation and failure of photovoltaic (PV) modules can be caused by changes in the mechanical properties of the polymeric components during the module lifetime. This paper introduces instrumented nanoindentation as a method to investigate the mechanical properties of module materials such as polymeric encapsulants. To this end, nanoindentation tests were carried out on ethylene vinyl acetate (EVA) surfaces, which have been separated from the glass panel. Two types of time-dependent indentation cycle modes, the time domain (creep mode) and frequency domain (dynamic mode) were performed to determine the viscoelastic behavior. For each mode, a corresponding model was applied to calculate the main mechanical properties. The general capability of nanoindentation as cross-linking determination method is investigated with the methodological advantages over bulk mechanical characterization methods. A large number of Glass/EVA/Backsheet laminates were built using different lamination conditions resulting in different degrees of curing. Both indentation modes indicate good modulus sensitivity for following the EVA crosslinking in its early stages but could not reliably differentiate between samples with higher EVA branching. Additional dynamic mechanical analysis (DMA) characterization was used as an established method to validate the indentation measurements. Both nanoindentation and DMA tensile mode produce similar quantitative viscoelastic responses, in the form of the damping factor parameter, demonstrated for three different frequencies at room temperature. A statistical study of the data reveals the advantages for the investigation of multilayer PV laminates by using nanoindenation as a surface method while also being applicable to field aged modules.

## 1. Introduction

Currently, there exists an increased interest from the photovoltaic (PV) industry in a fast and non-destructive method for the determination of the mechanical properties of solar encapsulant to ensure the encapsulation quality after lamination and the long-term stability. To implement this idea of a non-destructive approach, some studies have been proposed using ultrasonic detection [1,2], optical transmission [3] and mechanical indentation [4,5,6,7].

Dynamic mechanical analysis (DMA) has been established as suitable method to detect the viscoelastic properties of ethylene vinyl acetate (EVA) encapsulant [8]. However, the method has considerable drawbacks, including the required specimen geometry as well as a poor comparability of results obtained with different measurement parameters, which may prohibit the use of this technique. Alternatively, indentation testing shows a potential to quantitatively and non-destructively measure the gel content of low, medium, and highly cross-linked laminated solar encapsulants. The detection of the cured EVA’s viscoelastic response has been demonstrated previously by means of a compressive stress relaxation measurement. However, this mechanical testing was found to be representative only at elevated temperatures [7]. More recently, the instrumented indentation testing was used to distinguish different PV backsheets as freestanding polymer foils and quantify the effect of aging [9].

One of the most important influence factors for the EVA’s thermo-mechanical properties is the degree of curing (DoC). The polymer chains form a three-dimensional network during lamination through the thermolysis of peroxides, followed by a radical chain reaction, which crosslinks the individual polymer chains resulting in a certain DoC. An insufficient DoC can be the root cause for module degradation in the field, since under-cured EVA results in lower adhesion strength between layers [10]. Furthermore, the remaining unused crosslinking agents can trigger polymer degradation mechanisms involving acetic acid production [11,12,13]. On the other hand, an over-cured encapsulant might result in delamination and susceptibility to mechanical loads caused by a too high stiffness. Additionally, the EVA encapsulation plays a crucial role in the degradation of module performance. It has been demonstrated that highly-crosslinked EVA mediates thermal cycling (TC) stresses, while a low crosslinked EVA performed better under DH stresses. It is also observed that the level of power degradation depends on the degree of EVA crosslinking [14,15]. The two main parameters influencing the DoC are curing time [8,11,16] and curing temperature [14,15,16].

There are several methods to determine the DoC of EVA [8]. The Soxhlet method is a destructive and time-consuming method [17]. The correlations between the DoC and the other methods were validated using different analytical approaches such as (1) the detection of the actual crosslinks formed during lamination, for instance by means of IR or Raman spectroscopy [3,8,18,19], (2) the calculation of the residual amount of crosslinker present in the material after lamination, for instance by means of differential scanning calorimetry (DSC) [8,16,20,21] or (3) monitoring the changes of mechanical properties, for instance by means of scanning acoustic microscopy (SAM), laser vibrometry or DMA [8,10,20].

Compared to the previous techniques, the advantages of the instrumented nanoindentation are:

- Small sample size and reduced scale tests.
- Possibility to investigate multilayered material combinations (reduced sample preparation compared to DMA).
- Accurate and advanced tests for time-dependent materials like viscoelastic polymers.
- Spatial resolution allows to study the homogeneity of mechanical parameters along surfaces [22].
- Automated and Instrumented technique with good reproducibility.

**Figure 1:**
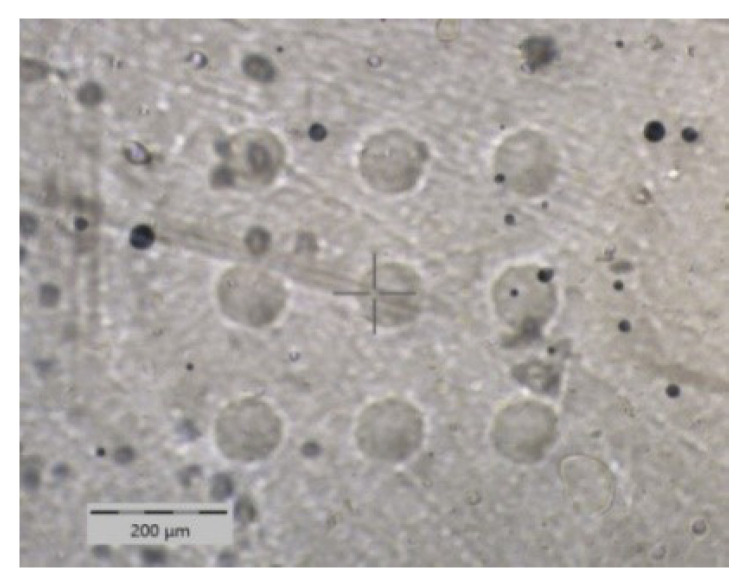
Optical microscopy image of indented EVA surface.

This paper introduces an advanced nanoindentation test including the time-dependent cycle modes for the quantification of the viscoelastic response of the EVA surface at the polymer-glass interface (Figure 1). To the best of our knowledge nanoindenation is used for the first time on EVA surface using time-dependent cycle modes. In this approach, first the elastic creep and viscoelastic sweep parameters are measured; then correlated to the EVA’s DoC as measured by the DSC method by using the exothermal peak from 110 to 190 °C. Furthermore, the viscoelastic properties as determined by the dynamic nanoindentation method are validated by DMA as an established technique.

## 2. Background

The instrumented indentation technique involves pressing an indenter of known geometry into the surface while both normal load and penetration depth are monitored. From the obtained force-displacement curve, the indentation hardness (HIT), elastic modulus (EIT) [23,24], viscoelastic properties of polymers [25,26] and other mechanical properties [27] can be obtained. The data analysis is performed automatically via Anton Paar’s indentation 8.0.26 software. This is an advantage compared to classical hardness measurements in which each imprint has to be measured separately with a microscope.

**Figure 2:**
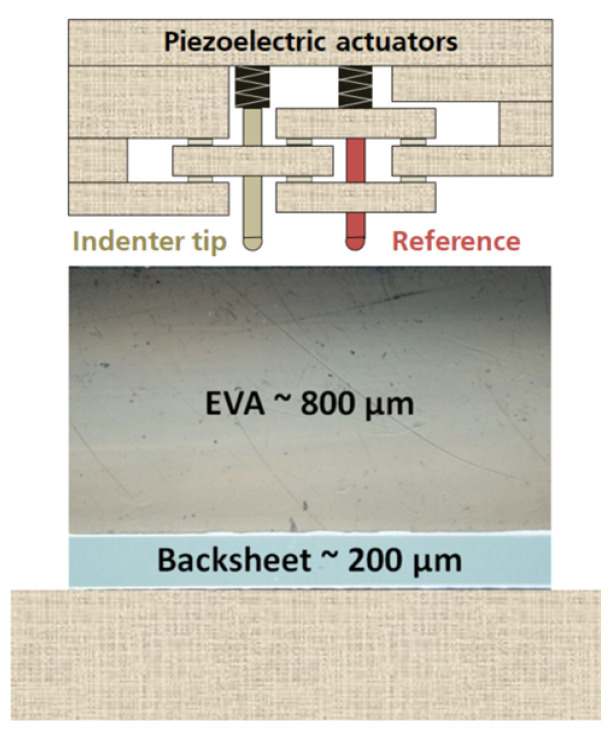
Illustration of the Ultra Nanoindentation Head Assembly applied to the multilayer EVA-BS film.

Figure 2 shows a schematic illustration of the indenter head, where a reference ball probe is used to monitor the surface of the sample continuously during the measurements using a piezoelectric actuator. This setup allows for an extremely low thermal drift during the indentation tests [28]. The thermal drift of the instrument is usually calculated and calibrated before the measurement [29,30].

The geometry of the indentation tip plays a critical role for the experiment. Depending on the mechanical properties of the material under test, differently shaped tips-usually made from diamond-can be used. The advantage of using a blunt indentation tip with low loads on time-dependent materials (polymers) is essentially the avoidance of plastic deformation of the sample. Therefore, the contact problem can be solved by Hertz equations, modified for the calculation of the viscoelastic properties of the polymer [31,32,33]. Moreover, the stresses in the indentation tests should be lower using blunt indenter geometry than a sharp tip [34]. In order to describe the creep and viscoelastic behavior of the material, two time-dependent indentation cycle modes are possible.

### 2.1. Creep Measurements: Ramp and Hold Indentation

**Figure 3:**
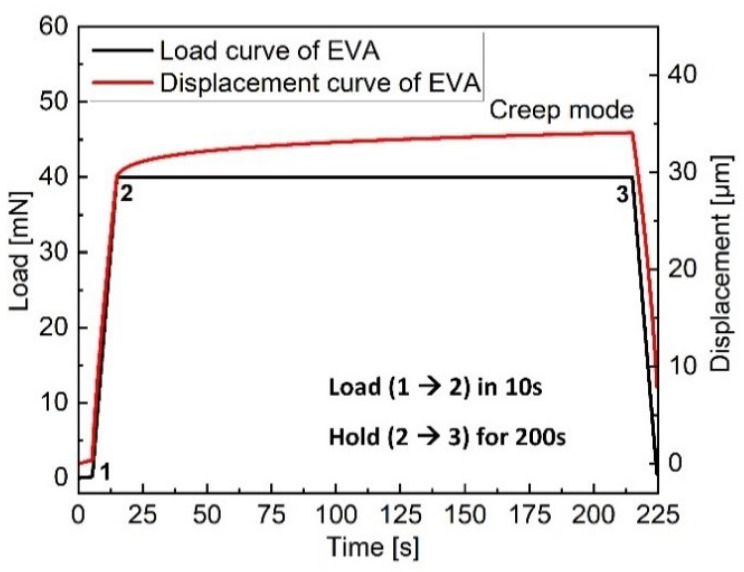
Creep load-displacement-time curves on the EVA surface obtained using a maximum load 40 mN, duration of creep 200 s.

Within the time domain, creep measurements are used to describe the long-term viscoelastic properties. The material is assumed to be linearly viscoelastic and therefore a step-like stress profile is recommended to express the influence of time [26]. The example cycle in Figure 3 consists of fast loading of 10 s up to 40 mN followed by a long-lasting hold of 200 s at constant force, during which the increase of depth (creep) is monitored. (see Table 1).

**Figure 4:**
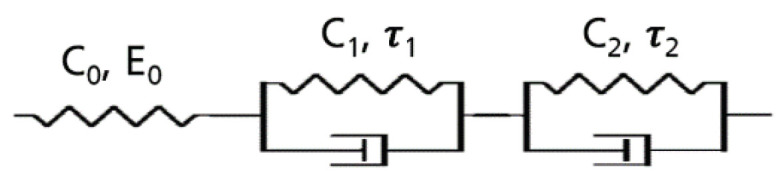
Schematic representation of two-mode Kelvin–Voigt mode.

This typical evolution of the EVA creep h(t) can be approximated by a model consisting of a spring with stiffness ($C_{j}$: compliances and $\tau {}_{j}$: retardation times) in series with two Kelvin-Voigt bodies (Figure 4) (see Equation (1)) [35]. The experimental data can be then fitted to the spherical indentation ramp–creep solutions to calculate the values of time-dependent elastic modulus in a Hertz contact model, where the elastic modulus is replaced with creep compliance as function of time [34,36,37].

(1) $${\left(h\left(t\right)\right)}^{m}=KP\left\{C_{0}+\Sigma C_{j}\left[1-\rho_{j}\exp(-\frac{t}{\tau_{j}}\right)\right]\}$$

where *m* is 3/2 for spherical indenter, *K* is $3/(4\sqrt{R})$, *P* is load and the term $\rho {}_{j}$ is the ramp correction factor [28,35].

At instantaneous time, the elastic modulus ($E_{0}$) is described in terms of all the coefficients ($C_{j}$: and $\tau {}_{j}$) (Equation (2)) and at infinite long time the relaxed elastic modulus ($E_{\infty }$) is described using only the elastic coefficient ($C_{0}$) (Equation (3)) [29,36,38]. The creep ration (CR) (Equation (4)) measures the extent of viscoelasticity, describing how much is the material creeping.

(2) $$E_{0}=E\left(0\right)=\frac{1}{J(0)}=\frac{1}{\left(C_{0}+\Sigma C_{j}\right)}$$

(3) $$E_{\infty }=E\left(\infty \right)=\frac{1}{C_{0}}$$

(4) $$CR=1-\frac{E_{\infty }}{E_{0}}$$

### 2.2. Dynamic Frequency Sweep: Sinus Indentation

During a sinus measurement, small load oscillations (amplitude and frequency) are controlled. This load generates displacement oscillations with a phase angle. When testing viscoelastic materials with the such a similar oscillatory methods, there is a lag (phase shift) δ in strain compared to force oscillations. The storage and loss moduli represent the elastic portion or stored energy (storage modulus $E^{\prime }$) and viscous part or dissipated energy (loss modulus $E^{\prime \prime }$) of the material [39]. They are defined by the Equations (5) and (6).

(5) $$E^{\prime }=\frac{\sigma_{0}}{\epsilon_{0}}\cos\delta$$

(6) $$E^{\prime \prime }=\frac{\sigma_{0}}{\epsilon_{0}}\sin\delta$$

where $\sigma_{0}$ and $\epsilon_{0}$ are stress and strain, respectively and δ is the phase shift between stress and strain [25]. The strain lag δ is often used in form of (tanδ), which is called the damping factor. This last describes the materials ability to disperse and absorb energy in relation to the stored potential energy in the material viscoelastic behavior of the polymers, which is defined by the following Equation (7).

(7) $$tan\delta =\frac{E\prime \prime }{E^{\prime }}$$

**Figure 5:**
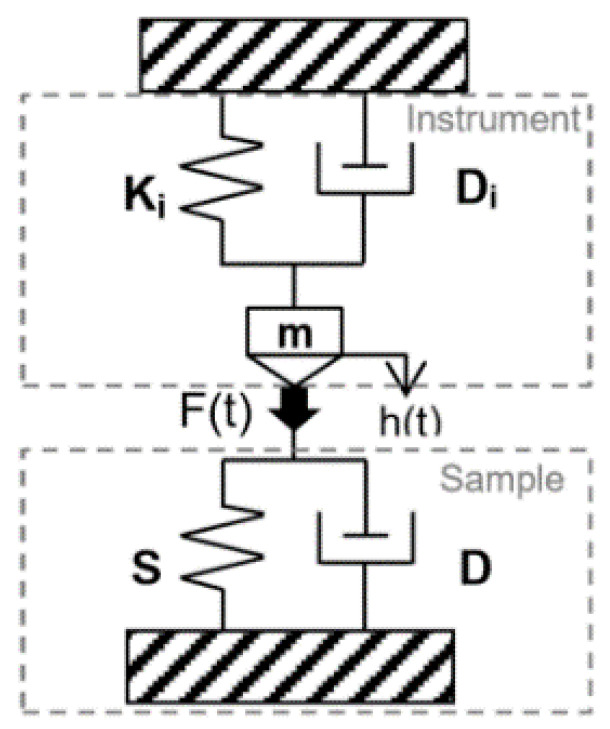
Dynamic mechanical model of viscoelastic material [42].

The dynamic mechanical testing method has also been applied to indentation testing. Several research papers summarize the implementation of the model and analysis of oscillatory indentation [40,41]. Within the limits of linear viscoelasticity (i.e., displacement amplitude is much smaller than the indentation depth), the indentation storage and indentation loss moduli (E′ and $E^{\prime \prime }$) are given in Equations (8) and (9) respectively.

(8) $$\frac{E^{\prime }}{1-\nu^{2}}=\frac{\sqrt{\pi }}{2\beta \sqrt{A_{p}}}\left(\frac{F_{0}}{h_{0}}\cos\delta +m\omega^{2}-K_{i}\right)$$

(9) $$\frac{E^{\prime \prime }}{1-\nu^{2}}=\frac{\sqrt{\pi }\omega }{2\beta \sqrt{A_{p}}}\left(\frac{F_{0}}{\omega h_{0}}\sin\delta -D_{i}\right)$$

where ν is the Poisson’s ratio, β describes a geometrical term, *A* is the projected contact area, $F_{0}$ and $h_{0}$ are force and displacement amplitudes and δ is phase shift between force and displacement. $K_{i}$ and $D_{i}$ are stiffness and damping coefficient of the instrument respectively, ω=2Πf where *f* is the Sinus frequency and *m* is the mass of the indenter. The oscillation properties of the instrument (stiffness, indenter mass, etc.) are determined during dynamic calibration procedure. This procedure is launched by the user and it is completely automatic. The viscoelastic response of the tested samples is modeled using a spring with stiffness *S* in parallel with a dashpot with damping factor *D*. Using the half amplitude of load and displacement signals, the measured phase angle and angular frequency of oscillation are used for the calculation of *S* and *D* (Figure 5). These two parameters are then used for the determination of the $\left(E^{\prime }\right)$ and the $\left(E^{\prime \prime }\right)$.

**Figure 6:**
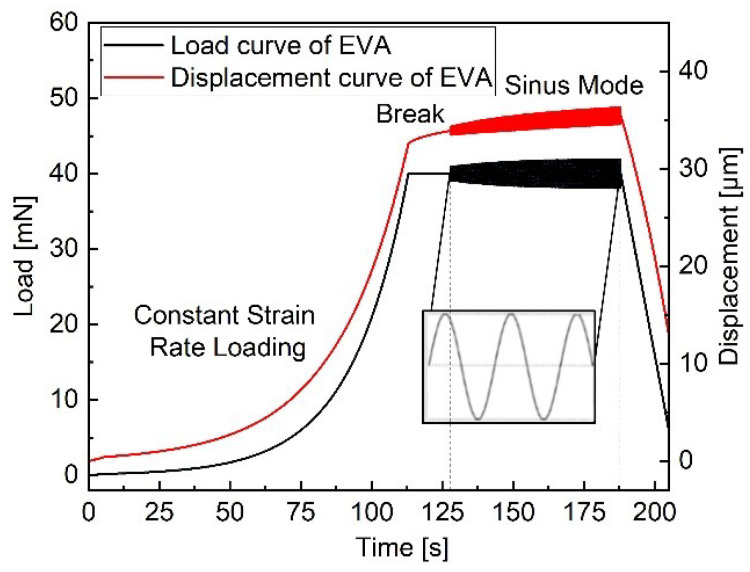
Dynamic load-displacement-time curves on the EVA surface obtained using a frequency sweep during the hold.

Figure 6 illustrates the load-displacement-time cycle used for the sinus indentation. To ensure similar loading conditions during measurements for all samples, a constant strain rate is used (up to 40 mN). The loading phase is followed by 15 s waiting time at constant load to obtain an equilibrium state. Oscillations are then applied at a fixed load for 65 s. The mean and standard deviation of storage and loss modulus at a given depth during the oscillations are then calculated. All dynamic measurements are performed at room temperature using the same indentation parameters as seen in Table 2.

## 3. Materials and Methods

### 3.1. Lamination and Sample Preparation

**Figure 7:**
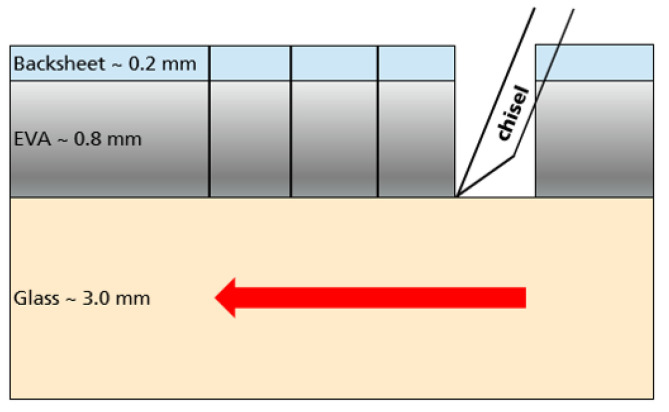
Schematic depiction of the investigated laminate during the sample preparation for nanoindentation and shear tests at DMA.

PV laminates (20 × 20 cm${}^{2}$) were prepared by laminating a layer of commercially available backsheets, 2 layers of EVA (PHOTOCAP^®^ 15580P/UF, Specialized Technology Resources, Inc., Enfield, CT, USA) as encapsulant and a transparent solar glass cover using 30 different lamination processes (ranging in curing temperature from 120 °C to 160 °C in 10 °C steps and curing time from 2 to 12 min in 2 min steps) in a Meier laminator Icolam 10/08. The glass-encapsulant interface was separated using a chisel type blade and allowed the extraction of 20 mm × 20 mm backsheet-encapsulant laminates. From these laminates 9 mm diameter samples were punched for DMA-shear tests and nanoindentation surface measurements (see Figure 7).

Simultaneously, samples for the DoC measurements by DSC [16] and DMA-tensile tests were laminated according to the following setup using the same lamination cycle: 1 Teflon based release sheet (50 µm), 2 layers of EVA (PHOTOCAP^®^ 15580P/UF, Specialized Technology Resources, Inc., Enfield, CT, USA), 1 Teflon based release sheet (50 µm) and a transparent solar glass cover (20 × 20 cm²). After removal of the EVA sheet from the release sheets, samples were punched using a 6 mm × 20 mm punch for DMA-tensile tests and 4 mm diameter punch for DSC measurements.

### 3.2. Differential Scanning Calorimetry (DSC)

Changes in the curing state of the EVA bulk were monitored by means of DSC after each lamination. The characterization was performed on a TA Instruments DSC Q200 system. The EVA layers were extracted from the laminates and prepared as described and placed in an aluminum crucible with punctured lid. Thermograms were recorded under constant nitrogen flow and at a heating rate of 10 °C/min from 25 °C to typically 250 °C. The reaction enthalpy was determined by the deviation from a linear baseline between 100 °C and 200 °C. The DoC was calculated using the enthalpy of the crosslinking reaction of the cured samples (Hsample) as well as an uncured sample (Huncured) as shown in Equation (10) [16].

(10) $$X\left(DoC\right)=1-\frac{H_{sample}}{H_{uncured}}$$

**Figure 8:**
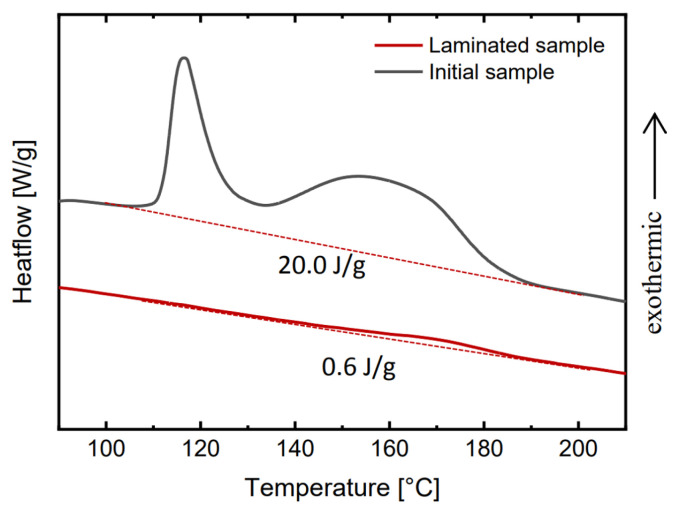
Typical DSC thermograms of uncured and laminated EVA.

Figure 8 shows two DSC exemplary thermograms where the enthalpies of partially cured EVA (($H_{sample}$) = 0.6 J/g) and uncured EVA (($H_{uncured}$) = 20.0 J/g) were deduced according to prevalent literature [8,16,20], resulting in a DoC of 96.8%. The complete results are discussed below in Section 4.1.

### 3.3. Nanoindentation

Changes in the viscoelastic properties of EVA surfaces were investigated using instrumented Nanoindenter machines manufactured by Anton Paar GmbH:

- The Ultra-Nanoindentation Testing (UNHT³) (Figure 2) with sphero-conical indenter tip geometry.
- The Nanoindentation Testing (NHT²) with flat punch-end conical indenter tip geometry.

In this work, we focus on Sphero-conical (a tip radius of 0.1 mm and an opening angle of 90° [26]) and flat punch-end conical (a tip diameter of 100 µm and an opening angle of 60°) blunt tip geometries i.e., for shallow indentations.

A distinct advantage of the flat punch geometry is that, even in the presence of creep behavior, the contact cannot change much, and hence, neither can the amplitude of the oscillation.

Tip calibration was performed on a fused silica reference sample from Anton Paar with a thermal drift ∼0 νm/N [39]. The samples prepared were measured at the EVA surfaces (glass-EVA interface) and outer backsheet surfaces. The samples require a flat surface with a very low surface roughness and must be supported underneath. The loading-holding-unloading parameters for both cycle modes were kept constant for all samples as summarized in Table 1 and Table 2, where a matrix of 9 measurements with a x and y spacing of 200 µm was performed (see Figure 1).

### 3.4. Dynamic Mechanical Analysis (DMA)

**Figure 9:**
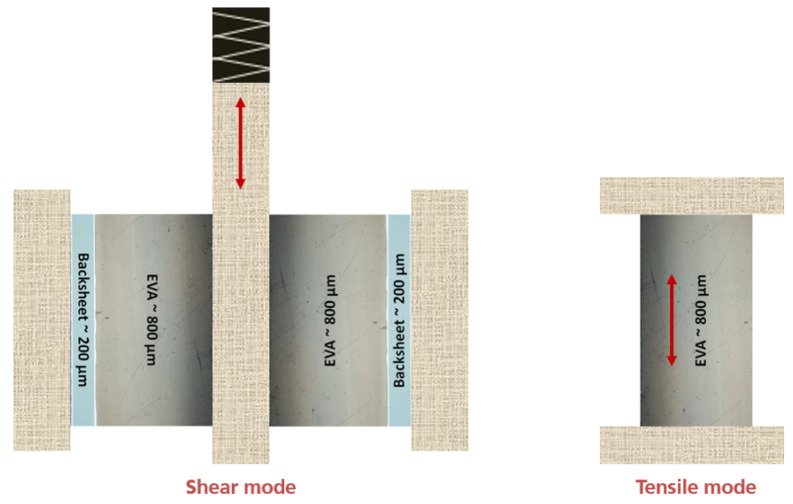
Schematic experimental setup DMA. The arrows represent the direction of the force excitation during measurement.

DMA is an established method for studying the viscoelastic behavior of EVA based PV encapsulants [8]. After each lamination, the polymer samples were measured by means of a Netzsch 242 E DMA for the determination of $E^{\prime }$, $E^{\prime \prime }$ and tanδ at room temperature at varying frequencies, using two modes:

- Shear mode (Figure 9): 3 samples (backsheet-EVA laminate) were prepared as described in Section 3.1.
- Tensile mode (Figure 9): 3 samples (EVA) were prepared as described in Section 3.1.

## 4. Results and Discussion

### 4.1. Crosslinking Determination by DSC

**Figure 10:**
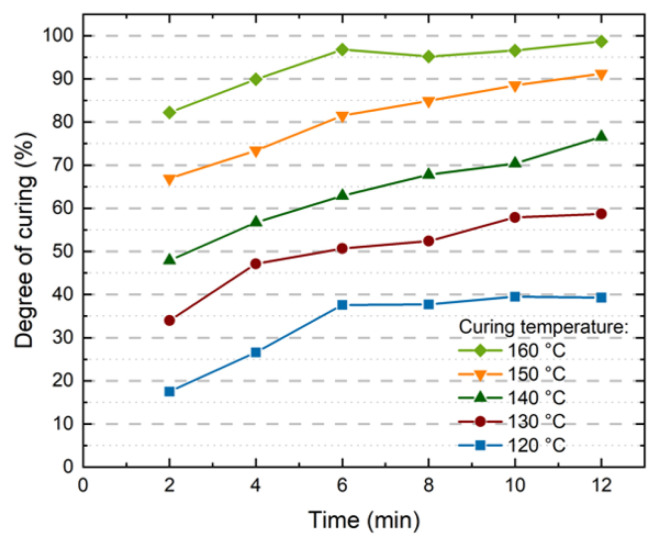
Correlation between the Degree of curing as measured by DSC of the processed 30 samples and the lamination parameters time and temperature.

Samples’ DoC were determined by DSC. For the comparison between indentation and DMA results, samples from the same 5 laminates with different lamination temperatures were measured. As seen in Figure 10 the different times and temperatures during the lamination process result in a range of DoC ranging from 17.5% up to 98.7%. The correlation between the lamination process and the curing of encapsulants has already been studied in detail [8]. In this publication the focus lies on the changes of the mechanical properties in the encapsulants. The variation of the DoC of one encapsulant allows for a first sensitivity and feasibility analysis of the methods for mechanical characterization to be investigated.

### 4.2. Creep Measurement Data to DoC Correlation

**Figure 11:**
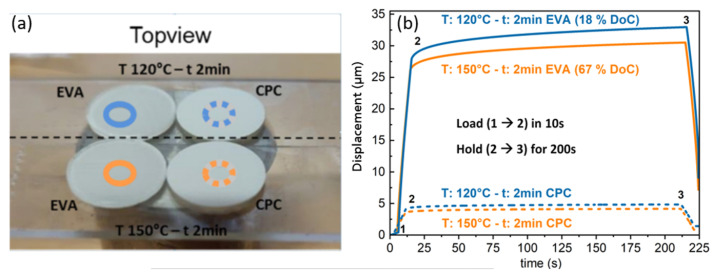
(**a**) Image of mounted samples with exposed surfaces of interest (EVA with linear circles and backsheet (CPC) with dashed circles) for nanoindentation tests. (**b**) Creep data on the same surfaces with 40 mN maximum load 40, 200 s duration of creep.

One approach of this study is to correlate EVA’s DoC as measured by DSC to the indentation creep of the EVA surface. To this end, comparative evaluations of creep response of the 30 laminates were undertaken using ramp-and-hold protocol as described in Section 2.1. Nanoindentation is a surface characterization method with a penetration depth of less than 0.5 µm, therefore the EVA-backsheet laminate samples were measured from both sides as shown in Figure 11a.

Creep curves in Figure 11b showed that highly cured samples with higher curing temperature (T = 150 °C) yield lower displacement from both surface sides, compared to lower curing temperature (T = 120 °C). These results indicate the possibility of detecting the EVA cross-linking response non-destructively from the backsheet via nanoindentation at room temperature. However, the influence of lamination conditions on the backsheet’s mechanical properties has not been considered. Therefore, only results from the EVA surface are discussed in this work, which can be interpreted quantitatively.

**Figure 12:**
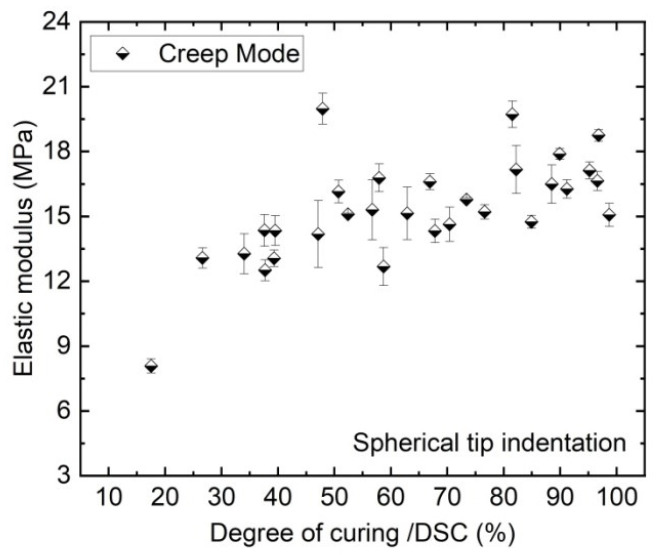
Data of elastic creep modulus (calculated from the creep curves) of the EVA surface as a function of 30 different DoCs as measured by DSC.

Figure 12 shows the creep elastic modulus of the EVA calculated from the experimental displacement-time curves and the numerical model of 30 samples with different DoCs. These creep measurements yielded a lower creep elastic modulus (∼8 MPa) for the lowest cured sample (T 120 °C–t 4 min) and a small increase of the modulus for degrees of crosslinking above 50%.

The creep modulus values obtained from the spherical indentation indicate a reduction of creep with the increasing of DoC. This change is significant for the most poorly crosslinked EVA. The increase of the modulus upon lamination is attributed to the formation of a three-dimensional polymer network. At around 50% DoC the modulus levels off. The overestimated modulus at around 20 MPa (∼50% DoC) could be caused by a tilting of the samples during the measurements.

### 4.3. Correlation of Dynamic Nanoindentation Data to DoC

**Figure 13:**
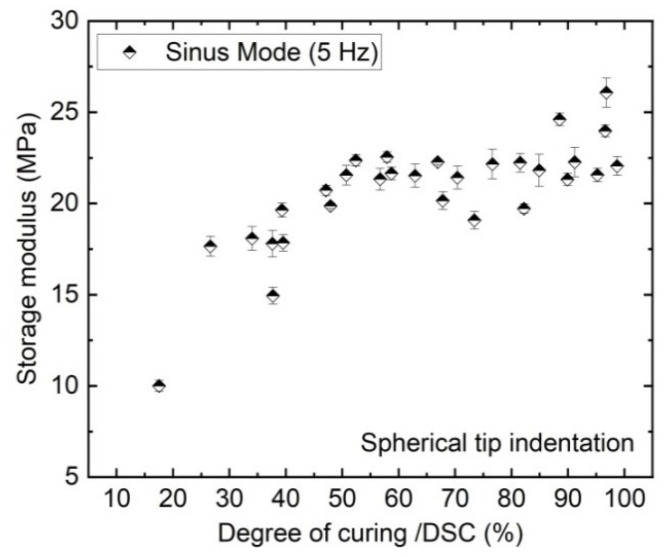
Storage modulus (calculated from the sine part of the dynamic curves) of the EVA surface as a function of 30 different DoCs as measured by DSC.

The storage modulus $E^{\prime }$ of the EVA describes the change of elastic properties of the polymer chains. Figure 13 shows $E^{\prime }$ of the EVA calculated from the sinusoidal part of the dynamic curve at 5 Hz as shown in Figure 6 and the numerical model in Figure 5 of the same 30 samples. Evaluated against the DoC, the E′ from the dynamic nanoindentation shows a similar relationship as for the creep elastic modulus shown in Figure 12: the modulus values increases strongly over the early phase of cross-linking, but shows a smaller increase at higher DoCs. Compared with the previously discussed results from creep measurements, the absolute values differ due to the change of measurement method and applied load.

Both nanoindentation approaches (creep and dynamic modes) possess a good modulus sensitivity for following the EVA crosslinking in its early stages, where additional entanglements lead to a modulus increase. In contrast, both approaches could not reliably differentiate between samples with higher DoCs [8]. At this DoC range, the polymer might be sufficiently crosslinked and almost all polymer chains are connected. If there are more radicals that result in even more crosslinks, the thermo-mechanical stability might not change sufficiently. In other words, it might be that the material shows no more detectable changes after crosslinking above a certain level. Other effects, such as a change molar mass, are possible; but the DoC change is most prominent during lamination due to the polymer formulation, which is why we focus on this parameter.

### 4.4. Correlation of Tensile DMA Measurement Data to DoC

**Figure 14:**
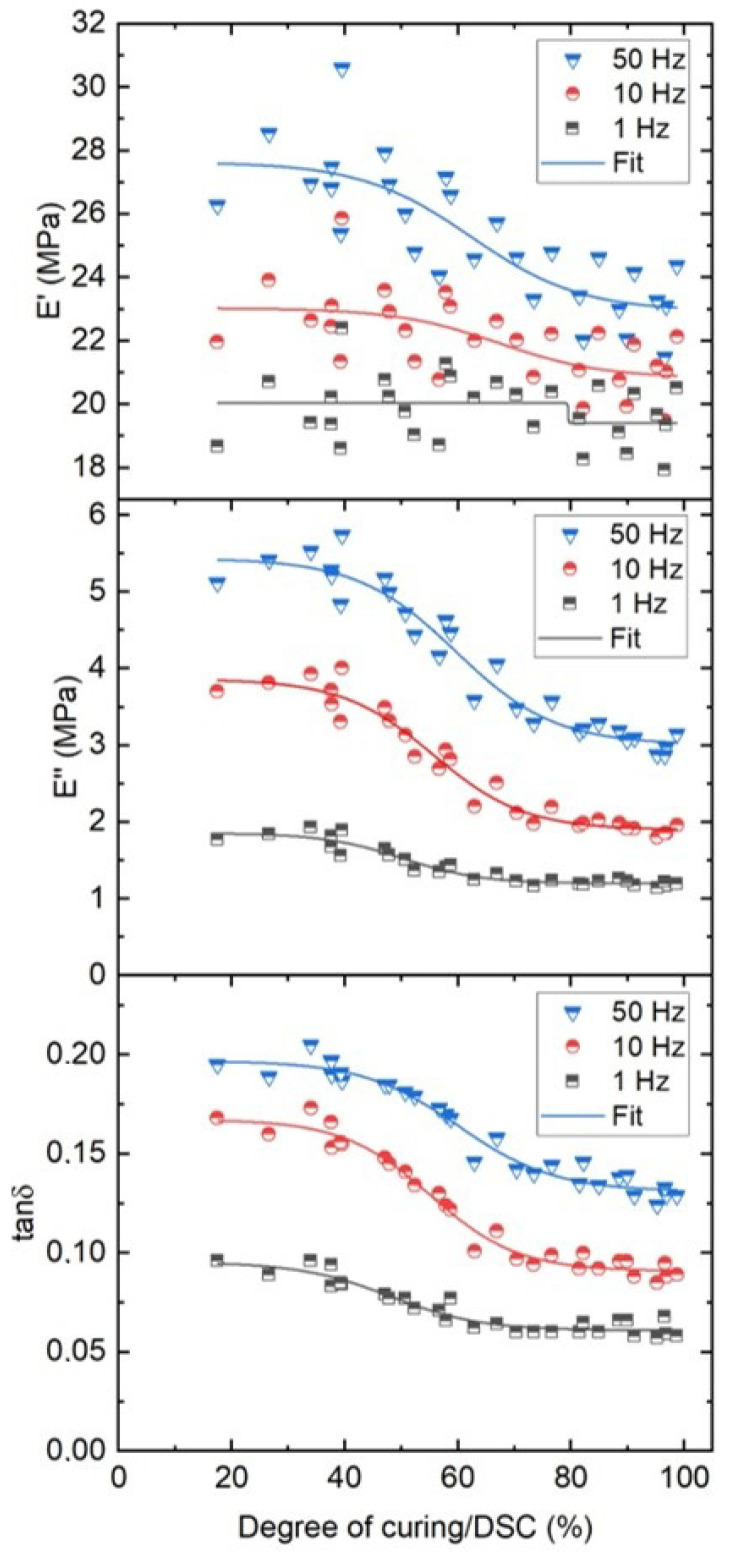
Storage modulus (top), Loss modulus (mid) and Damping factor (bottom) calculated from tensile-DMA of the EVA bulk as a function of 30 different DoCs.

In this section we compare the results of the DMA measurements in tensile mode to the DoC of EVA to investigate which material properties are sensitive enough for a correlation. DMA measurements with tensile mode are performed on the EVA bulk using three different frequencies (1, 5 and 50 Hz). All 30 samples were measured once and the resulting values for $E^{\prime }$, $E^{\prime \prime }$ and tanδ are presented in Figure 14.

The storage modulus is highly fluctuating for these samples independent of the used frequency. However, for higher frequencies a clear trend can be observed where a higher DoC results in a lower storage modulus. For both values of $E^{\prime \prime }$ and tanδ a better correlation and less uncertainty can be observed. This indicates that the changes observed due to crosslinking are mostly impacting the polymers ability to disperse energy.

As a comparative study, a fitting model to quantify the effects of the different DMA parameters on the DoCs is proposed here. The presented fitting model is calibrated on the experimental data to extract the coefficient of determination $R^{2}$ parameter as summarised in Table 3. The fits are plotted with the measurements in Figure 9. The $R^{2}$ values confirm the poor correlation of the $E^{\prime }$ to the DoC and a better fit of $E^{\prime \prime }$ and tanδ, especially at lower frequencies.

### 4.5. Nanoindentation vs. DMA-Test-Retest Reliability

Following these measurements, 5 samples of varying DoCs were chosen to investigate the test–retest reliability. Due to the low response of DMA measurements at 1 Hz the frequency range was increased to 5 Hz, 50 Hz and 100 Hz. Subsequently, dynamic nanoindentation flat punch indentation measurements were also performed at the EVA surface of the laminates using three different frequency sweeps (5 Hz, 40 Hz and 100 Hz).

**Figure 15:**
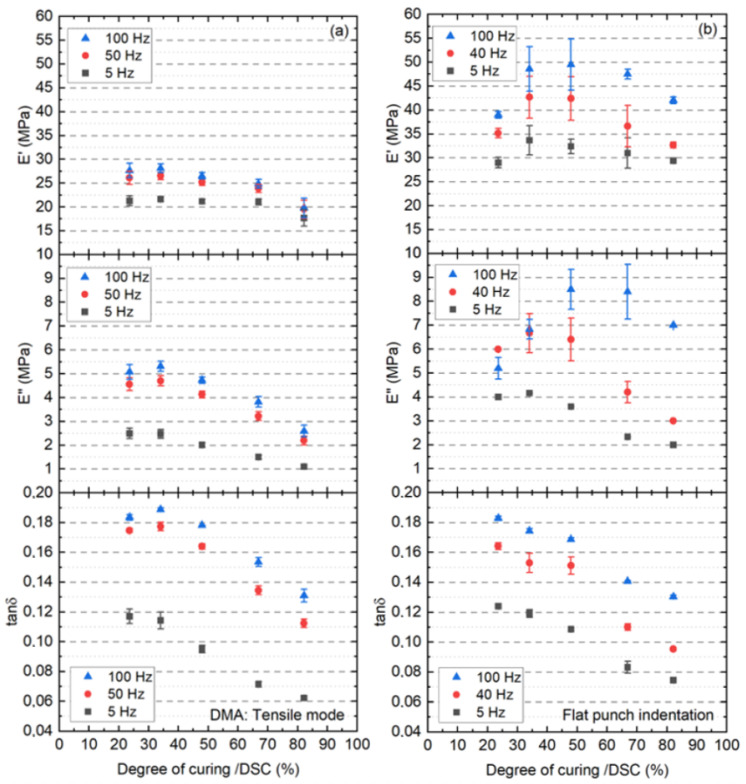
Storage modulus (top), loss modulus (mid) and damping factor (bottom) calculated from DMA tensile mode of the EVA bulk (**a**) and nanoindentation dynamic mode of the EVA surface (**b**) as a function of 5 different DoCs.

Figure 15 illustrates a direct comparison of $E^{\prime }$, $E^{\prime \prime }$ and tanδ for the EVA in relation to DoC for each tested frequency as determined by DMA-tensile mode and nanoindentation at room temperature, where each point is an average of three measurements for the DMA and nine for the nanoindentation measurements at a given frequency, respectively. As observed before and especially for the nanoindenation, $E^{\prime }$ clearly shows a poor correlation to DoC. More precisely, the nanoindentation $E^{\prime }$ value increases slightly with the EVA’s DoC (as shown earlier for the spherical indentation). This different behavior of the $E^{\prime }$ between using both techniques can be explained by the different volume of the material response. On the contrary, the correlation quality of $E^{\prime \prime }$ to DoC start to increase, especially at 5 Hz.

Furthermore, a correlation between tanδ and DoC is observed for all frequencies used. We would like to point out that the observed decrease in tanδ corresponds to an increase of crosslinking bonds density, which also increases the molecular weight of the material restricts their molecular mobility. The strongest decrease of tanδ was found between the values corresponding to DoCs of 48% and 67%.

A second notable result is that the two methods (dynamic nanoindentation and DMA-tensile mode) yield comparable tanδ despite the different volume of the material response: The DMA in tensile mode represents the response of the EVA bulk, while the nanoindentation represents the response of the EVA surface, where the volume considered is several orders of magnitude smaller than in the DMA. This is surprising when comparing results obtained with techniques using different measuring material dimensions.

**Figure 16:**
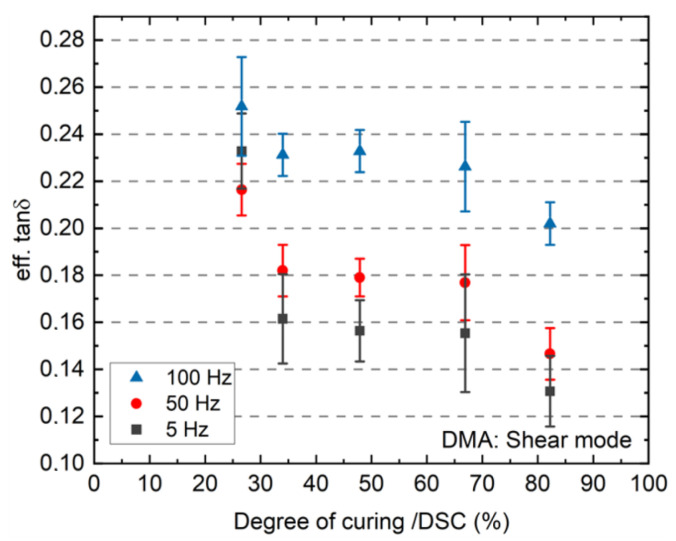
Damping factor calculated from DMA shear mode of EVA-backsheet laminates as function of 5 different DoCs.

Figure 16 shows the evolution of the effective damping factor ($tan\delta_{eff}$) of the material combination backsheet-EVA calculated from DMA-shear mode measurements in relation to the EVA’s DoC from the chosen laminates with five different lamination temperatures. These results show overestimated damping factors with high error bars which could originate from the additional influence of the backsheet as well as the relatively high aspect ratio of the EVA for a shear experiment. A sample with a ratio of 0.8 mm thickness to 10 mm diameter needs high forces to achieve a reasonably measurable deflection. Therefore influences of plastic deformation or increased errors due to the low deflection might occur. The thickness of ∼800 µm was chosen to represent a geometry typically found in commercial PV modules. Nevertheless, the same trend of $tan\delta_{eff}$ was observed independently of the frequency.

Table 4 summarizes the analytical methods discussed earlier for the test-retest reliability of EVA viscoelastic properties. For each method/approach, information is given about the mechanical material response, applicability to the components after sample preparation, measured viscoelastic variable which can be assessed and the test-retest reliability for correlation quality of the mechanical parameters to DoC. To obtain a quantitative comparison of the tanδ measured at 100 Hz (taken as an example) using the three approaches, the coefficient of variation (CV) is presented here, where it represents the ratio of the standard deviation to the mean values of each measured sample. This is a useful statistic for comparing different methods or measured values regarding their repeatability. A lower CV for repeated measurements using a certain method indicates a more reliable method. As can be seen in Table 4, the CV of tanδ measured from nanoindentation shows the smallest value.

## 5. Conclusions and Outlook

In this work we introduced nanoindentation as method that allows to investigate multilayer materials with highly reduced effort as well as simpler sample preparation. Towards this end, we introduced two different nanoindentation approaches (creep measurements and dynamic sweep measurements) and correlated the results with established methods (DSC and DMA).

As shown above, nanoindenation method allows the measurement of mechanical properties of the EVA encapsulation materials with the application of extremely low load. Specifically the loss modulus $E^{\prime \prime }$ and tan(δ) shows promise due to a stronger correlation with the DoC of EVA than the storage modulus $E^{\prime }$.

The nanoindentation method was validated by conventional DMA: Measurement results from both nanoindenation and DMA tensile mode proved to produce nearly identical damping factors, despite the different volume of the material response. Furthermore, the dynamic nanoindentation exhibited a favorable test-retest reliability for tan(δ) compared with the other investigated methods.

The method provides the possibility of the application of nanoindentation to investigate the encapsulant’s viscoelastic changes from field-exposed PV modules or modules after accelerated aging tests. In the future, the nanoindenation will be applied as a non-destructive method for the detection of the EVA cross-linking response measured through the backsheet at room temperature. Further steps should include the development of a the correlation equation between the extent of the damping factor used as a critical parameter and module performance parameters such as the power output. Such a correlation is not directly obvious, but indirect effects of EVA aging that influence the module performance could be corrosion due to acetic acid formation or cell cracks due to embrittlement. We hope to have laid the groundwork for an easier investigation of the material properties inside PV modules to obtain a better understanding of the crosslinking progress, especially at the onset of the PV module lamination. This should contribute to shortening PV module lamination times as well as optimizing the encapsulant formulation.

## Figures and Tables

**Table 1:** Indentation parameters for creep mode.

Loading/unloading rate	0.004 s${}^{-1}$
Maximum load	40 mN
Holding time	200 s

**Table 2:** Indentation parameters for creep mode.

Constant strain rate	0.05 s${}^{-1}$
Maximum load	40 mN
Break before Sinus	15 s
Sinus duration	65 s
Sinus frequency	5 Hz, 40 Hz, 100 Hz
Amplitude	4 A

**Table 3:** Extracted coefficient of determination $R^{2}$ for the fit functions shown in Figure 14.

Parameter	Frequency (Hz)	$R^{2}$
	50	0.67
storage modulus (E${}^{\prime }$)	10	0.39
	1	0.10
	50	0.95
Loss modulus (E${}^{\prime \prime }$)	10	0.96
	1	0.92
	50	0.96
Damping factor (tanδ)	10	0.97
	1	0.91

**Table 4:** Comparative summary of the investigated approaches and their correlation to DoC.

Characterization	Material	Applicability to	Measured	Coefficient
Method-Mode	Response	PV Components	Variable	of Variation **
NI-dynamic	EVA surface	laminates *	tanδ	0.4%
DMA-shear	laminate bulk	laminates	$tan\delta_{eff}$	8.4%
DMA-tensile	EVA bulk	EVA sheets	tanδ	1.9%

* laminates: Backsheet-EVA films. ** The Degree of variation is taken from samples with 67% of DoC.

## Data Availability

Not applicable.
